# Lymph Node Invasion by Melanoma Cells Is Not Required for the Induction of Incomplete Differentiation by Tumor‐Specific CD8+ T Cells

**DOI:** 10.1002/cnr2.70145

**Published:** 2025-02-10

**Authors:** Kristian M. Hargadon, Travis B. Goodloe, Stephen L. Woodall

**Affiliations:** ^1^ Hargadon Laboratory, Department of Biology Hampden‐Sydney College Hampden‐Sydney Virginia USA

**Keywords:** cancer, CD8 T cell dysfunction, lymph node invasion, melanoma, tumor immunity

## Abstract

**Background:**

Lymph node invasion by cancer cells is a poor prognostic factor and is often associated with anti‐tumor CD8+ T cell dysfunction. In this study, we investigated the role of lymph node invasion by melanoma cells in the induction of incomplete differentiation by tumor antigen‐specific CD8+ T cells.

**Aims:**

We aimed to determine whether lymph node invasion by melanoma cells is required for this specific form of anti‐tumor CD8+ T cell dysfunction.

**Methods and Results:**

We assessed lymph node invasion by the B16‐F1 and D5.1G4 murine melanoma cell lines and evaluated tumor antigen‐specific CD8+ T cell responses to these melanomas in the context of tumor‐free versus tumor‐involved lymph nodes. We demonstrate that CD8+ T cells recognizing antigen from established melanomas fail to acquire effector function, regardless of whether the tumor is stable or progressive. This CD8+ T cell dysfunction arises in the context of both tumor‐involved and tumor‐free lymph nodes draining established melanomas.

**Conclusions:**

Lymph node invasion by melanoma cells is not required for the induction of incomplete CD8+ T cell differentiation. These data and their implications for strategies to enhance CD8+ T cell responses against poorly immunogenic melanomas are discussed herein.

## 1 Introduction

Melanoma is the most lethal form of skin cancer due to its propensity to metastasize to several vital organs, including the brain, lungs, liver, and other visceral organs [1]. Additionally, one of the first sites of melanoma metastasis during tumor progression is the regional lymph nodes (LN), and 5‐year survival rates for patients with LN metastases are significantly lower than those for patients whose tumors have not invaded regional LN at the time of diagnosis [2, 3]. Among patients presenting with LN metastases, tumor burden within melanoma‐involved LN has also been shown to correlate negatively with survival [4, 5, 6]. Importantly, LN invasion by melanoma is often associated with tumor‐specific CD8+ T cell dysfunction [7, 8, 9, 10, 11, 12], but it remains unclear whether such dysfunction depends on LN invasion by tumor cells or whether it can arise independently of, and prior to, cancer cell metastasis to regional LN.

While checkpoint blockade therapy has proven useful in protecting CD8+ T cells from anergy and exhaustion, a third type of immune dysfunction that does not appear to be related to co‐inhibitory signaling is also frequently observed in melanoma patients and is characterized by incompletely differentiated CD8+ T cells that have proliferated but failed to acquire effector functions [7, 9, 13]. This preterminally differentiated phenotype has been observed in antigen (Ag)‐specific CD8+ T cells recovered from both tumor‐involved lymph nodes (TILN) and tumor‐free lymph nodes (TFLN) of melanoma patients [9]. Despite the significance of these observations, however, insights into the induction of this form of tumor immune dysfunction are limited in the context of clinical settings, where it is unknown for any given patient how long either a tumor or its associated anti‐tumor immune response has been established at the time of study. Such limitations necessarily preclude the assessment of naturally‐induced anti‐tumor T cell responses immediately following exposure to native tumor Ag, and thus it remains unclear from the clinical work to date whether the presence of incompletely differentiated T cells in TFLN results from suboptimal activation of T cells at this site or, alternatively, reflects the presence of T cells that had previously been exposed to tumor Ag within TILN before recirculating to TFLN within the same nodal basin. As insight into factors that contribute to the induction of incomplete CD8+ T cell differentiation may inform strategies to prevent or overcome this type of anti‐tumor immune dysfunction, we wished to investigate whether LN invasion by melanoma is necessary to prevent effector differentiation of CD8+ T cells in tumor‐draining LN.

Previous work in the B16 murine melanoma model has recapitulated the incompletely differentiated phenotype of tumor Ag‐specific CD8+ T cells often observed in melanoma patients—in a lung colonization model, CD8+ T cells responding within the context of TILN draining the lungs exhibit robust proliferation but do not differentiate into functional effectors [14]. This failure of CD8+ T cells to fully differentiate was found to arise in TILN whether tumor Ag was directly presented by invading melanoma cells or cross‐presented by host Ag‐presenting cells (APC), though CD8+ T cells did acquire effector function if Ag recognition occurred in TFLN at the time of tumor challenge. While these prior findings highlight a correlation between incomplete differentiation and LN metastasis, CD8+ T cell differentiation in response to established tumors that have yet to invade regional LN was not previously investigated, and it has remained unclear whether the failure of tumor‐specific CD8+ T cells to fully differentiate into function effectors is dependent on LN invasion by cancer cells. Therefore, in this study we have employed the B16‐cOVA model and a newly developed model of LN noninvasive melanoma (D5.1G4‐cOVA) to investigate whether LN metastasis is required for the induction of incomplete CD8+ T cell differentiation. We show that in response to both highly aggressive and poorly tumorigenic melanomas, CD8+ T cells within tumor‐draining LN fail to undergo effector differentiation following recognition of Ag derived from established tumors, regardless of whether tumor cells have invaded the draining LN at the time the response is initiated.

## 2 Materials and Methods

### 2.1 Mice

Female C57Bl/6 mice and OT‐I RAG2^−/−^ mice were purchased from Taconic Biosciences (Germantown, NY). Female C57Bl/6 Thy1.1 congenic mice were purchased from The Jackson Laboratory (Bar Harbor, ME). All mice were used between 8 and 12 weeks of age and weighed in the range of 18–23 g. Experiments were approved by the Hampden‐Sydney College Animal Care and Use Committee and were performed in accordance with regulatory standards and guidelines outlined in the Guide for the Care and Use of Laboratory Animals.

### 2.2 Established Cell Lines

D5.1G4 murine melanoma cells were a generous gift of Dr. Jerry Neiderkorn (University of Texas Southwestern Medical School). B16‐F1 murine melanoma cells were purchased from the American Type Culture Collection (ATCC) in Manassas, VA. These tumor cell lines were grown in RPMI‐1640 medium supplemented with 2 mM L‐glutamine, 2 g/L glucose, and 2 g/L sodium bicarbonate (Thermo Fisher Scientific), as well as 10% fetal bovine serum (Premium Select, Atlanta Biologicals, Norcross, GA). The JAWSII cell line (ATCC) is a GM‐CSF‐dependent, C57Bl/6 bone marrow‐derived DC line that grows as a mixture of adherent and suspension cells. JAWSII cells were grown in HyClone Iscove's Modified Dulbecco's Medium (Thermo Fisher Scientific) supplemented with 10% fetal bovine serum (Premium Select, Atlanta Biologicals), 4 mM L‐glutamine, HEPES, 0.1 mM β‐mercaptoethanol (Sigma, St. Louis, MO), 1 mM sodium pyruvate (Sigma), and 5 ng/mL recombinant murine GM‐CSF (Thermo Fisher Scientific). All cells were passaged at 80%–90% confluence.

### 2.3 Generation of Cytoplasmic Ovalbumin (cOVA)‐Expressing and tdTomato‐Expressing Tumor Cell Lines

B16‐F1 and D5.1G4 melanoma cells were transfected with a pEF6 plasmid encoding cytoplasmic chicken ovalbumin using Attractene Transfection Reagent (Qiagen, Germantown, MD) according to the manufacturer's protocol. Transfected cells were cultured for 24 h in the absence of antibiotics and then grown under blasticidin selection (10 μg/mL during generation). Following blasticidin‐induced death of all cells in an untransfected control group, the surviving polyclonal cell transfectants were then cloned by limiting dilution, and expanded clones were screened for expression of H‐2K^b^‐OVA_257_ complex expression and recognition by CFSE‐labeled (1 μM) OT‐I cells in a co‐culture system. In some cases, tumor cells were pre‐treated for 24 h with 20 ng/mL recombinant murine IFNγ prior to analysis. Blasticidin selection of the established cOVA‐expressing lines was continued at a maintenance concentration of 5 μg/mL throughout the duration of the studies.

Similarly, we generated tdTomato‐expressing variants of B16‐F1 and D5.1G4 by stably transfecting these cell lines with a pEF1α‐tdTomato expression vector (Takara Bio USA, San Jose, CA). Cells transfected as described above were grown under G418 selection (800 μg/mL), and surviving cells were cloned by limiting dilution. Clones were expanded under ongoing G418 selection and screened for stable tdTomato expression by flow cytometric analysis of fluorescence in the FL2 channel on an Accuri C6 flow cytometer (BD Biosciences, San Jose, CA).

### 2.4 Antibodies and Reagents

The following anti‐mouse monoclonal antibodies were purchased from BioLegend (San Diego, CA): anti‐H‐2K^b^ APC (AF6‐88.5), anti‐H‐2K^b^‐OVA_257_ APC (25‐D1.16), LEAF‐purified anti‐CD3ε (145‐2C11), anti‐CD4 Alexa Fluor 488 (RM4‐5), anti‐CD8α PE (53–6.7), anti‐CD25 PE (A18246A), anti‐CD69 APC (H1.2F3), anti‐Thy1.2 PerCP‐Cy5.5 (53–2.1), anti‐IFNγ PE (XMG1.2), and anti‐FOXP3 Alexa Fluor 647 (MF‐14). CFSE was purchased from eBioscience (San Diego, CA). Peptide comprising residues 257–264 of chicken ovalbumin (SIINFEKL) was purchased from AnaSpec (Fremont, CA). Recombinant murine IFNγ protein, a LEGENDplex Mouse Th Cytokine Panel (13‐plex) kit, and a True‐Nuclear Transcription Factor Buffer Set were all purchased from BioLegend.

### 2.5 Tumor Challenge and Adoptive Transfer Experiments

For tumor challenge experiments, 4 × 10^5^ tumor cells were injected in a volume of 0.2 mL sterile, endotoxin‐free 1X PBS (Teknova, Hollister, CA) intravenously via the lateral tail vein. Mice were euthanized on the indicated days, lungs were harvested for counting melanoma nodules under a dissecting microscope, and lung‐draining paratracheal LN were harvested to assess tumor cell invasion or anti‐tumor CD8+ T cell responses.

To obtain OVA_257_‐specific CD8+ T cells for adoptive transfer experiments, single cell suspensions were obtained by homogenization of pooled spleen and LN (inguinal, axillary, brachial, mesenteric, para‐aortic, and cervical) of OT‐I RAG2^−/−^ mice. Cells were passed through a 70 μm nylon mesh cell strainer, red blood cells were lysed with RBC Lysis Buffer, and cells were washed with MACS buffer before passage through another cell strainer. CD8+ T cells were then enriched by negative selection using a CD8+ T Cell Isolation Kit from Miltenyi Biotec Inc. (Auburn, CA) according to the manufacturer's protocol. Enriched populations were consistently > 95% CD8+, and 2.0 × 10^6^ cells were adoptively transferred intravenously into Thy1.1 congenic tumor‐bearing mice via the lateral tail vein.

### 2.6 Detection of Melanoma Cells in Tumor‐Draining LN

To assess in vivo invasion of lung‐draining paratracheal LN by melanoma cells, expression of the gene encoding the melanocyte differentiation antigen tyrosinase‐related protein 2 (*Trp2* gene) was measured in single‐cell suspensions derived from homogenized LN. RNA was isolated from cells using the RNeasy Mini Kit from Qiagen. Following RNA extraction, samples were treated with Amplification Grade DNase I (Life Technologies, Grand Island, NY) to digest any residual DNA, and RNA was quantified using an Epoch Spectrophotometer (BioTek, Winooski, VT). Reverse transcription was performed with 500 ng RNA using random primers and SuperScript II Reverse Transcriptase from Life Technologies. Quantitative real‐time PCR was performed on a StepOne Plus Real‐Time PCR System (Thermo Fisher Scientific) with 2 μL of the resulting cDNA using Thermo Fisher Scientific's TaqMan Universal Master Mix II with UNG and TaqMan FAM‐MGB Gene Expression Assays specific for *Trp2* (Assay ID Mm01225584_m1) or *Gapdh* (Assay ID Mm99999915_g1) as a housekeeping gene control. Relative gene expression was calculated by normalizing *Trp2* gene expression against that of the *Gapdh* housekeeping gene.

To demonstrate the limit of detection for melanoma cells within lung‐draining paratracheal LN populations using this approach, B16‐F1 melanoma cells were also serially diluted and seeded at concentrations of 1, 10, 100, and 1000 cells per individual whole LN single‐cell suspension. RNA was isolated from these populations for quantitative real‐time RT‐PCR analysis as described above.

In related experiments, lung‐draining paratracheal LN were harvested from tumor‐bearing mice and homogenized into single‐cell suspensions. These preparations were passed through 70 μm cell strainers to remove debris, and cells were cultured ex vivo for 3 days in tumor media supplemented with 100 U/mL penicillin and 100 μg/mL streptomycin (ATCC). The outgrowth of tumor cells from these cultures was monitored on an Olympus CK2 inverted microscope and documented with an INFINITY1‐3C digital camera using INFINITY CAPTURE software (Lumenera Corporation, Ottawa, Ontario, Canada).

LN invasion experiments were also performed in separate animals with tdTomato‐expressing B16‐F1 and D5.1G4 cell lines. Lung‐draining paratracheal LN were harvested from mice at various time points following intravenous challenge with 4 × 10^5^ B16‐F1‐tdTomato or D5.1G4‐tdTomato cells. Homogenized LN were passed through 70 μm cell strainers to remove debris, and single cell suspensions of entire LN populations were then analyzed by flow cytometry for the presence or absence of tdTomato‐expressing cells.

### 2.7 Isolation and Stimulation of OT‐I Cells From Tumor‐Bearing Mice

Lung‐draining paratracheal LN were harvested at various time points following adoptive transfer of OT‐I cells into tumor‐bearing mice. For intracellular cytokine staining assays, paratracheal LN populations were stimulated for 5 h in media containing 1 μg/mL GolgiPlug (BD Biosciences) and 1X Monensin Solution (BioLegend) with JAWSII stimulator cells that had been pulsed with 1 μg/mL OVA_257_ peptide. Cells were fixed in 2% paraformaldehyde for 15 min at room temperature and resuspended in FACS Buffer (PBS supplemented with 2% fetal bovine serum, 2 mM EDTA, and 0.02% NaN_3_) for Fc blocking at 4°C for 5 min. Surface staining was performed with the indicated monoclonal antibodies for 30 min at 4°C in the dark. For intracellular cytokine staining, cells were permeabilized with Perm/Wash (BD Biosciences), and nonspecific intracellular antibody binding was blocked by resuspending and incubating cells in Perm/Wash containing 5% normal goat serum (Jackson ImmunoResearch Laboratories Inc., West Grove, PA) for 20 min on ice in the dark. Following this incubation, anti‐IFNγ antibody was added directly to the cells for 30 min at 4°C in the dark. Labeled cells were detected by flow cytometry using an Accuri C6 flow cytometer (BD Biosciences) and were analyzed using BD Accuri C6 software.

In related experiments, we also enriched Thy1.2+ (CD90.2+) OT‐I cells from the paratracheal LN of Thy1.1 congenic tumor‐bearing mice by magnetic activated cell sorting using CD90.2 microbeads from Miltenyi. Three consecutive rounds of positive selection yielded samples of at least 95% Thy1.2+ purity. These enriched OT‐I cells were then plated into 96‐well plates that had been coated the day before with 1 μg/mL of LEAF Purified anti‐CD3ε antibody for ex vivo restimulation. Culture supernatants were collected 24 h post‐restimulation for analysis of cytokine release using a LEGENDplex Mouse Th Cytokine Panel (13‐plex) kit.

### 2.8 Analysis of Regulatory T Cells Within Tumor‐Draining Lymph Nodes

LN harvested from tumor‐bearing mice were homogenized and stained for surface expression of CD4 and CD25 before being fixed and permeabilized for FOXP3 staining according to the manufacturer's protocol supplied with BioLegend's True‐Nuclear Transcription Factor Buffer Set.

### 2.9 Statistical Analysis

Values are expressed as the mean ± standard deviation (SD) and differences among indicated groups were analyzed using unpaired *t* tests. All experiments were conducted at least 3 independent times, with 2–3 mice per group within each replicate. Sample sizes were based on achieving a statistical power of 0.8, with *α* = 0.05 and *β* = 0.2. A value of *p* ≤ 0.05 was considered significant and is represented in graphs by *. **p ≤ 0.01, ***p ≤ 0.001, ****p ≤ 0.0001.

## 3 Results

### 3.1 Differential Outgrowth and Lymph Node Metastasis by the B16‐F1 and D5.1G4 Murine Melanoma Cell Lines

**FIGURE 1:**
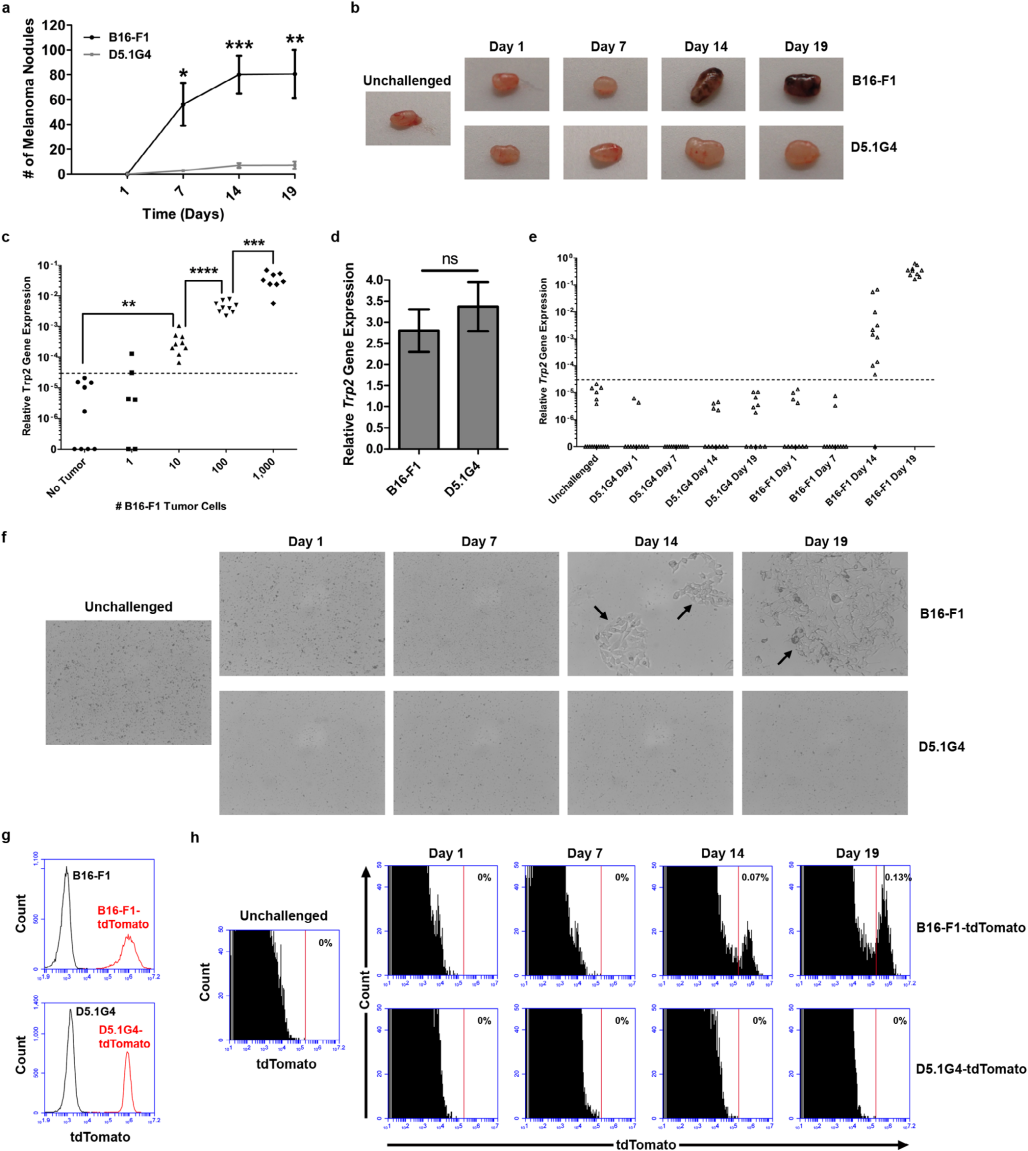
Differential outgrowth and LN invasion by B16‐F1 versus D5.1G4 melanoma. (a) C57Bl/6 mice were challenged intravenously with 4 × 10^5^ tumor cells as indicated, and lung nodules were counted on the indicated days, highlighting the progressive outgrowth of B16‐F1 as compared to its less tumorigenic D5.1G4 counterpart. Data are plotted as the average from 3 independent experiments, each with 2 mice per group, and error bars reflect the SD of the mean. (b) Lung‐draining paratracheal LN were harvested from B16‐F1 or D5.1G4 tumor‐bearing mice on the indicated days for visible inspection of pigmentation. (c) B16‐F1 tumor cells were seeded as indicated into single‐cell suspensions derived from separate, individual homogenized paratracheal LN (~1e6 cells per individual LN), and *Trp2* gene expression was evaluated and plotted relative to *Gapdh* gene expression. *Trp2* gene expression was consistently detected above background when as few as 10 melanoma cells were seeded into whole LN populations. Pooled data from 3 independent experiments, each with 2–4 replicates per condition are plotted. (d) Comparison of relative *Trp2* gene expression in the B16‐F1 and D5.1G4 cell lines. (e) C57Bl/6 mice were challenged with 4 × 10^5^ tumor cells intravenously, and lung‐draining paratracheal LN were harvested on the indicated days. Assessment of *Trp2* gene expression within these whole LN populations revealed that B16‐F1, but not D5.1G4, melanoma cells invade regional LN draining tumor‐bearing lung tissue. Pooled data from 5 independent experiments, each with 2–4 mice per group are shown. (f) In other experiments, single‐cell suspensions were generated from the paratracheal LN of tumor‐bearing mice and cultured for 3 days. LN invasion by melanoma cells was then assessed by evaluating whether tumor cells (indicated by bold arrows) could be grown out of these LN cultures, as is shown for LN harvested from B16‐F1 tumor‐bearing mice. Representative images from 3 independent experiments, each with 2 mice per group are shown. (g) Overlay plots of wild‐type (black histogram) versus tdTomato‐expressing (red histogram) melanoma cell lines. (h) Evaluation of LN invasion by tdTomato‐expressing tumor cells in mice bearing B16‐F1‐tdTomato or D5.1G4‐tdTomato melanomas for the indicated number of days confirmed invasion of lung‐draining paratracheal LN by only the tdTomato‐expressing variant of B16‐F1. Data are representative of 3 independent experiments, each with 2 mice per group, as summarized in Table 1.

B16‐F1 is a well‐characterized, highly aggressive murine melanoma cell line, and CD8+ T cell responses initiated against Ag derived from this melanoma at late stages of tumor progression are characterized by robust proliferation but minimal effector function [14], a phenotype similar to that observed in CD8+ T cells recovered from many metastatic melanoma patients. D5.1G4 is a chemically mutated variant of B16 melanoma [15], and we have previously documented differential outgrowth between it and B16‐F1 in a lung colonization model [16]. A more thorough kinetic analysis of the outgrowth of these tumors revealed progressive growth of B16‐F1 in the lungs over time (Figure 1a). Although the number of melanoma nodules appears to stabilize by Day 14 post‐challenge, lesions continue to grow in size after this time and begin taking over the lung tissue, making it difficult to distinguish individual lesions at later stages of tumor outgrowth, and mice typically had to be euthanized due to signs of distress by 21 days post‐challenge with this tumor. On the other hand, although small D5.1G4 nodules are detectable in lung tissue within 7 days of tumor challenge, these nodules do not increase in number or size over the timeline we investigated (*p* > 0.05 for Day 7 vs. Day 14 and for Day 7 vs. Day 19), making this cell line an excellent model for studying stable, nonprogressing disease.

In addition to the observed differences in outgrowth between B16‐F1 versus D5.1G4 tumor cells, we also observed pigmentation within the lung‐draining paratracheal LN at later stages of tumor progression in B16‐F1 tumor‐bearing mice (Figure 1b), suggesting LN invasion by B16‐F1, but not D5.1G4, melanoma cells. Indeed, previous work has shown that B16‐F1‐derived Ag can be directly presented by tumor cells to CD8+ T cells within the lung‐draining paratracheal LN at 14 days post‐intravenous tumor challenge [14]. However, the kinetics of LN invasion by this tumor have not been previously investigated, and even though D5.1G4 outgrowth does not progress rapidly in lung tissue, it is unknown whether these cells might still be able to invade regional LN at low levels. Therefore, we developed an assay to assess melanoma cell invasion of the lung‐draining paratracheal LN by measuring in whole LN populations the expression of the *Trp2* gene that encodes tyrosinase‐related protein 2, an enzyme uniquely expressed in melanoma cells/melanocytes, where it is involved in the melanin deposition pathway. As shown in Figure 1c, *Trp2* gene expression is completely undetectable in paratracheal LN cell populations of many C57Bl/6 mice, and the low background signal that was detected in some mice was used to set a threshold for detection of bona fide *Trp2* expression. Seeding of these LN populations with varying numbers of B16‐F1 melanoma cells demonstrated the high sensitivity of this assay. In some cases, we were able to detect *Trp2* gene expression above the background threshold when only a single tumor cell was seeded into cell suspensions from homogenized LN. Although detection of a single melanoma cell by this measure was inconsistent, significant *Trp2* gene expression was observed 100% of the time when 10 tumor cells were seeded into these paratracheal LN populations, and we observed the expected ~10‐fold exponential increase in *Trp2* gene expression in these populations when 100 and then 1000 tumor cells were added to the suspensions. Importantly, the *Trp2* gene is expressed at similar levels in B16‐F1 and D5.1G4 melanoma cells (Figure 1d), yet in tumor‐challenged mice we observed expression of this gene only in paratracheal LN draining lungs bearing B16‐F1 tumors. Detection of *Trp2* gene expression within these LN above the background threshold was not observed in mice bearing B16‐F1 tumors through the first 7 days following tumor challenge but was observed in all mice by 14 days post‐challenge and continued to increase over time, indicating an increase in tumor burden at this site during tumor progression. On the other hand, no significant *Trp2* gene expression was observed in the paratracheal LN of mice bearing D5.1G4 melanomas at any time point (Figure 1e), providing evidence that this tumor does not invade the lung‐draining LN within the time frame we have studied.

To further demonstrate that B16‐F1, but not D5.1G4, melanoma cells invade regional LN, we validated these *Trp2* gene expression studies with a more direct evaluation of tumor cell infiltration of the lung‐draining paratracheal LN. Specifically, we cultured single‐cell suspensions of paratracheal LN harvested from tumor‐bearing mice for 3 days to assess whether tumor cells could be grown out of these populations. As shown in Figure 1f, tumor cells could be cultured out of LN draining the lungs of mice bearing 14‐ and 19‐day‐old B16‐F1 tumors. Consistent with our *Trp2* gene expression assay, this approach revealed melanoma cells in the lung‐draining LN of 100% of mice by 14 days of B16‐F1 tumor challenge, whereas D5.1G4 melanoma cells could not be grown out of the paratracheal LN at any time (Table 1, top), even when the ex vivo culture period was extended to 10 days (Figure S1).

**TABLE 1:** Lymph node invasion by melanoma cells over time.

Melanoma cell outgrowth from tumor‐draining lymph nodes				
	Day 1	Day 7	Day 14	Day 19
B16‐F1	0/6	0/6	6/6	6/6
D5.1G4	0/6	0/6	0/6	0/6

Detection of tdTomato‐expressing melanoma cells in tumor‐draining LN				
	Day 1	Day 7	Day 14	Day 19
B16‐F1‐tdTomato	0/6	0/6	6/6	6/6
D5.1G4‐tdTomato	0/6	0/6	0/6	0/6

*Note:* (Top) C57Bl/6 mice were challenged intravenously with B16‐F1 or D5.1G4 melanoma cells, and the lung‐draining paratracheal LN were harvested on the indicated days post‐tumor challenge. Lymph node single cell suspensions were cultured for 3 days and observed for the outgrowth of tumor cells. Numbers indicate the proportion of mice from which tumor cells could be recovered from draining LN. These data are pooled from 3 independent experiments with 2 mice per group. (Bottom) C57Bl/6 mice were challenged intravenously with B16‐F1‐tdTomato or D5.1G4‐tdTomato melanoma cells, and the lung‐draining paratracheal LN were harvested on the indicated days post‐tumor challenge. LN single cell suspensions from entire LN populations were analyzed by flow cytometry, and numbers indicate the proportion of mice from which tdTomato‐expressing tumor cells were detected. These data are pooled from 3 independent experiments with 2 mice per group.

Finally, we engineered stable tdTomato‐expressing variants of B16‐F1 and D5.1G4 melanoma cells (Figure 1g) so that we could detect potential LN invasion by these cells in vivo. As shown in Figure 1h, when we harvested the paratracheal LN of mice bearing B16‐F1‐tdTomato or D5.1G4‐tdTomato tumors for the indicated days and assessed them for the presence or absence of tdTomato‐expressing cells by flow cytometry, we observed these cells only in the LN of mice bearing 14‐day‐old and 19‐day‐old B16‐F1‐tdTomato tumors. Consistent with our ex vivo LN culture experiments, we observed tdTomato‐expressing cells in LN draining these advanced B16‐F1‐tdTomato tumors in 100% of the mice bearing such tumors (Table 1, bottom). Collectively, these data highlight the differential potential for LN invasion exhibited by the B16‐F1 and D5.1G4 melanoma cell lines.

### 3.2 A Model System for Investigating the Induction of Tumor‐Specific CD8+ T Cell Responses in Tumor‐Free Versus Tumor‐Involved Lymph Nodes

**FIGURE 2:**
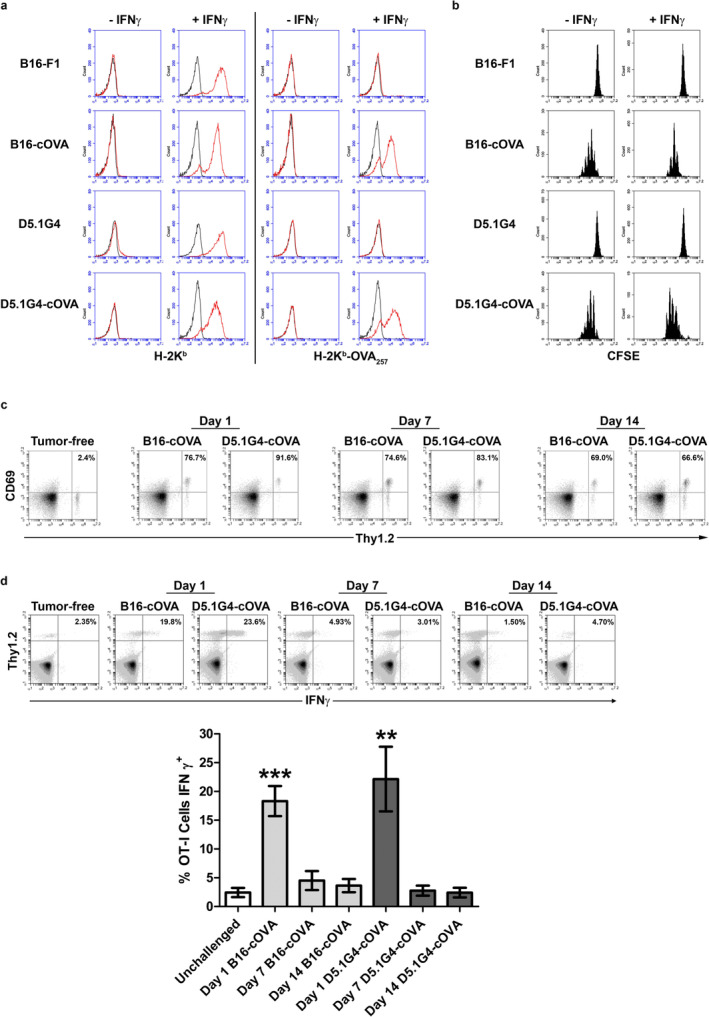
Induction of incomplete tumor Ag‐specific CD8+ T cell differentiation in tumor‐free and tumor‐involved LN. (a, b) B16‐cOVA and D5.1G4‐cOVA melanoma cells were left untreated or were pre‐treated for 24 h with IFNγ and then assayed for expression of H‐2K^b^ and H‐2K^b^‐OVA_257_ complexes as well as for recognition by CFSE‐labeled OT‐I cells in co‐culture for 4 days, demonstrating both experimental and biological detection of the OVA_257_ epitope on cells transfected with our expression vector. (c) Thy1.2+ OT‐I cells were adoptively transferred into C57Bl/6 Thy1.1 congenic mice that had been challenged intravenously with 4 × 10^5^ of the indicated tumor cells 1, 7, or 14 days previously. Lung‐draining paratracheal LN were harvested 24 h post‐adoptive transfer, and single‐cell suspensions were stained for flow cytometric analysis of CD69 expression as a readout of antigen recognition. Plots are gated on CD8+ lymphocytes, and percentages in the upper right corner indicate the percentage of Thy1.2+ OT‐I cells that are CD69+. Representative plots from 1 of 3 independent experiments, each with 2 mice per group are shown. (d) Thy1.2+ OT‐I cells were adoptively transferred into C57Bl/6 Thy1.1 congenic mice that had been challenged intravenously with 4 × 10^5^ of the indicated tumor cells 1, 7, or 14 days previously. Lung‐draining paratracheal LN were harvested 5 days post‐adoptive transfer, and single‐cell suspensions were restimulated with OVA_257_‐pulsed JAWSII cells for 5 h to assess effector differentiation by intracellular IFNγ staining. Significant IFNγ production was observed in OT‐I cells responding to recently injected tumor cells, but not well‐established tumor nodules in the lung, regardless of whether melanoma cells from these established tumors invaded regional LN. Plots are gated on lymphocytes, and percentages in the upper right corner indicate the percentage of Thy1.2+ OT‐I cells that are IFNγ+. Representative plots from 1 of 3 independent experiments, each with 2 mice per group are shown. Data from the 3 independent experiments are graphed as the average with error bars reflecting standard deviation of the mean. For statistical analysis, each group was compared against the unchallenged control group using an unpaired *t*‐test.

Based on the differences in LN metastasis between the B16‐F1 and D5.1G4 melanomas, these cell lines are useful tools for investigating how LN invasion influences the induction of anti‐melanoma immune responses in tumor‐draining LN. To this end, we generated cytoplasmic ovalbumin‐expressing variants of these cell lines (B16‐cOVA and D5.1G4‐cOVA) so that we could evaluate OVA_257_‐specific CD8+ T cell (OT‐I) responses to a tumor‐specific Ag in the context of TFLN versus TILN. First, we characterized cloned transfectants for expression of both H‐2K^b^ and H‐2K^b^‐OVA_257_ complexes. While neither the wild‐type nor the transfected variants of B16‐F1 and D5.1G4 cells expressed these molecules at a level detectable by flow cytometry under standard culture conditions, pre‐treatment with IFNγ induced H‐2K^b^ expression on all cell lines and H‐2K^b^‐OVA_257_ expression specifically in the transfected B16‐cOVA and D5.1G4‐cOVA cells (Figure 2a). Importantly, both B16‐cOVA and D5.1G4‐cOVA were recognized by OT‐I cells, as we observed CFSE dilution in labeled OT‐I cells that had been cultured with these Ag‐expressing tumors but not with their non‐Ag‐expressing wild‐type counterparts (Figure 2b). Indeed, OT‐I cells proliferated in response to both B16‐cOVA and D5.1G4‐cOVA even when these cells were not pre‐treated with IFNγ. These data indicate that while H‐2K^b^‐OVA_257_ complexes are not expressed on these tumor cells at high enough levels in the steady state to be detected by the less‐sensitive flow cytometric assay, they are expressed at levels sufficient for recognition by OT‐I cells in their natural state.

Previous work has demonstrated that the OVA_257_ Ag from B16‐cOVA can be directly presented to OT‐I cells by tumor cells that have infiltrated regional LN. This Ag can also be cross‐presented to OT‐I cells by host APC, both prior to and following LN invasion by the tumor [14]. Although our previous in vitro analyses demonstrated that OT‐I cells are capable of recognizing OVA_257_ on D5.1G4‐cOVA, Ag‐specific CD8+ T cell responses to this tumor have not been previously investigated in vivo. Therefore, we challenged Thy1.1 congenic C57Bl/6 mice intravenously with either B16‐cOVA or D5.1G4‐cOVA and then adoptively transferred Thy1.2+ OT‐I cells into these mice at various stages of tumor progression. Despite the differences in tumor outgrowth and LN metastasis exhibited by these tumors, we observed equivalent upregulation of the early activation marker CD69 on OT‐I cells in response to both melanomas at all stages of tumor progression analyzed (Figure 2c). Specifically, tumor Ag from D5.1G4‐cOVA was presented to OT‐I cells in the context of TFLN at times when B16‐derived Ag was presented in either TFLN (Day 1 and Day 7) or TILN (Day 14). We therefore took advantage of these models as tools to study tumor‐specific CD8+ T cell responses over time to a progressing, LN‐invasive melanoma versus a stable, LN‐noninvasive melanoma.

### 3.3 Lymph Node Invasion by Melanoma Is Not Required for the Induction of Incomplete Tumor Ag‐Specific CD8+ T Cell Differentiation

Previous studies in the B16‐cOVA model have shown that CD8+ T cells responding to Ag within the context of TILN proliferate but fail to acquire effector function, whereas those responding within TFLN to cross‐presented Ag from recently injected tumor cells differentiate into IFNγ‐secreting effectors [14, 17]. However, it is unclear whether the presence of tumor cells within draining LN accounts for this difference or whether the effector differentiation of CD8+ T cells observed early after tumor challenge reflects a difference in responsiveness to recently injected cells versus those associated with an established tumor. To address these issues, we adoptively transferred OT‐I cells into Thy1.1 congenic C57Bl/6 mice bearing 1‐, 7‐, or 14‐day‐old B16‐cOVA or D5.1G4‐cOVA tumors so that we could evaluate the differentiation of tumor Ag‐specific CD8+ T cells shortly after tumor challenge (Day 1) as well as in response to established tumors in the context of both TFLN (Day 7 B16‐cOVA, Day 7 and Day 14 D5.1G4‐cOVA) and TILN (Day 14 B16‐cOVA). Consistent with previous reports [14, 17], a significant fraction of OT‐I cells isolated 5 days after transfer into hosts bearing 1‐day‐old B16‐cOVA tumors were capable of secreting IFNγ following a brief ex vivo restimulation, whereas very few of these cells differentiated into IFNγ‐secreting effectors when responding within TILN of mice bearing 14‐day‐old B16‐cOVA tumors (Figure 2d). Interestingly, we found that even within the context of TFLN draining established 7‐day‐old B16‐cOVA tumors, OT‐I cells failed to acquire IFNγ‐secreting effector function. Importantly, this effect was not specific to the progressing B16‐cOVA tumor, as OT‐I cells responding within the context of TFLN to established D5.1G4‐cOVA (both 7‐ and 14‐day‐old tumors) failed to fully differentiate into IFNγ‐secreting effectors as well. Finally, while it was previously shown in the B16‐cOVA model that dysfunctional OT‐I cells responding to this tumor do not develop into IL‐4‐ or IL‐10‐producing cells [14], we confirmed these findings for OT‐I cells responding to D5.1G4‐cOVA, and we found that OT‐I cells responding to both tumors also failed to acquire IL‐5‐, IL‐9, or IL‐17‐secreting functionality (data not shown). Therefore, the lack of type 1 effector activity in CD8+ T cells responding to these established melanomas reflects incomplete differentiation, rather than an alternate program of differentiation. Our data are the first to demonstrate that LN invasion by melanoma cells is not necessary to drive the induction of this incompletely differentiated phenotype in tumor‐specific CD8+ T cells, and we show that this phenotype arises in CD8+ T cells responding to established melanomas regardless of whether disease is progressive or stable.

## 4 Discussion

Herein we show for the first time that LN invasion by melanoma cells is not necessary to drive induction of an incompletely differentiated phenotype in tumor‐specific CD8+ T cells, and we highlight incomplete differentiation as a unique form of tumor immune dysfunction that may arise early during the course of tumor progression. Despite the significance and potential implications of these findings, which we discuss in detail below, our study has two limitations that we wish to acknowledge. First, our work involves a transplantable model of murine melanoma, which does not reflect the natural progression of disease following melanocytic transformation. Furthermore, because we employed an intravenous tumor challenge approach to promote melanoma lung colonization, our experimental design bypasses key biological aspects of the metastatic cascade that are required for melanoma cells to migrate from primary skin lesions to lung tissue. However, although lung metastasis can be more accurately recapitulated in spontaneous and inducible models of metastatic melanoma [18, 19], to date it remains challenging to monitor the early stages of T cell activation against specific tumor Ag in such systems the way we have in our study. Moreover, we do note that despite inducing artificial lung colonization in our system, we have focused our study on CD8+ T cell differentiation within the lung‐draining LN, where tumor cells must naturally metastasize from melanoma lesions in the lung, and our experimental design does allow us to monitor T cell differentiation at this site at very defined stages of tumor progression.

We also recognize that because our study focuses on the differentiation of adoptively transferred CD8+ T cells specific for a foreign Ag, the response of T cells that we observe in our study is not impacted by mechanisms of self‐tolerance that indeed influence anti‐tumor immune reactivity to many naturally occurring tumor‐associated Ag. Our work is therefore more representative of the T cell reactivity that might be expected to occur against an oncoviral or tumor neoantigen, both of which remain useful targets of anti‐tumor immune responses. The fact that we observe incomplete differentiation of T cells even against such a strong, foreign tumor Ag in our study highlights the relevance of this form of T lymphocyte dysfunction as a potential barrier to the success of anti‐tumor immunity.

In addition to our current and prior work on this form of immune dysfunction in various models of melanoma, Schietinger et al. have also recently documented similarly dysfunctional tumor‐specific T cells in an inducible model of liver cancer, where they evaluated the effector activities of T cells in pre‐malignant liver tissue shortly after tumor induction. In their model, T cells were transferred into mice prior to tumor initiation and then followed over time at various stages post‐tumor induction. Although T cell dysfunction also arose early during the course of tumor progression in that model, it was found to depend on persistent antigenic stimulation in the tumor‐bearing liver [20]. In contrast, our study evaluated CD8+ T cell responses following transfer into mice already bearing tumor in the lungs, and T cells were harvested for analysis after a consistent period of Ag exposure, regardless of whether tumor cells had recently been injected or lung nodules/LN metastases had already become established. The fact that we recovered functional effector T cells after transfer into 1‐day tumor‐bearing mice reveals that the T cell dysfunction we observed in 7‐ and 14‐day tumor‐bearing mice could not have arisen simply from persistent antigenic stimulation, as T cells were exposed to tumor Ag for 5 days in all cases, regardless of the time at which they were transferred. Moreover, it is unlikely that tumor Ag load influenced the outcome of T cell stimulation in our model, as we observed the same dysfunctional phenotype whether T cells were responding to established tumors of the very slow‐progressing D5.1G4 melanoma or the rapidly progressing B16‐F1 melanoma. While we cannot discount the possibility that tumor Ag load and chronic antigenic stimulation might further impair T cell function and survival beyond the first 5 days of their initial differentiation in our model, particularly as both factors are known to be important in shaping the overall quality of T cell responses to tumors, the design of our experiments is such that these factors cannot be solely responsible for the induction of the incompletely differentiated phenotype we observe.

Consistent with our observations, Horton et al. recently found in an orthotopic mouse model of NSCLC that tumor‐specific CD8+ T cells primed in the lung‐draining LN also undergo a dysfunctional differentiation program characterized by proliferation that is decoupled from acquisition of effector function [21]. Interestingly, however, this dysfunctional phenotype was observed only in T cells reactive against orthotopic lung tumors and not in T cells responding to established B16 melanoma lung nodules. Though it remains unclear why tumor‐specific differences were observed by Horton et al. and why the quality of anti‐melanoma responses specifically differ across our two studies, it is possible that differences in the tumor Ag employed in our distinct models contribute to these discrepancies. Indeed, others have reported heterogeneity in CD8+ T cell responses directed against tumor‐derived SIYRYYGL (the model Ag utilized by Horton et al.) and SIINFEKL (the model Ag investigated in our current study) [22], and it may be that such differences are further compounded by factors specific to particular tumor cell types, such that Horton et al. observed different outcomes for T cells responding to melanoma versus NSCLC. Importantly, we do note that previous work on T cell reactivity against established B16 melanoma has documented incomplete differentiation in CD8+ T cells responding not only to the model neoantigen SIINFEKL but also to an endogenously processed Ag derived from the melanocyte differentiation protein tyrosinase (Tyr_369_) [14], a well‐characterized and naturally occurring human tumor Ag.

Our data suggest that the effector differentiation of CD8+ T cells that occurs following recognition of Ag from recently injected (1‐day‐old) tumor cells is likely artificial, perhaps as a result of the coagulative necrosis that is known to occur in these cells shortly after intravenous challenge [23]. Previous work has shown that tumor Ag presentation at this time occurs only by cross‐presentation [14], and an artificially‐induced immunogenic tumor cell death immediately after tumor challenge may therefore stimulate host APC that are capable of driving effector CD8+ T cell differentiation. Once surviving tumor cells have become established, however, it is possible that such APC stimulation does not occur, meaning that cross‐presentation of Ag at later stages from established tumor may take place in the absence of other stimulatory signals that are necessary to promote effector T cell differentiation [24]. Indeed, we have found that soluble factors derived from B16‐F1 (and to a lesser extent D5.1G4) actually interfere with dendritic cell (DC) maturation and activation [25]. A similar lack of T cell stimulation is likely to accompany direct presentation of Ag by poorly immunogenic tumor cells that have invaded regional LN, and previous work in the B16‐cOVA model has shown that incomplete CD8+ T cell differentiation arises following either direct or cross‐presentation of Ag from late‐stage tumors [14].

Although we favor suboptimal T cell stimulation as a driver of incomplete differentiation in our model, we cannot rule out the possibility that CD8+ T cell differentiation is directly suppressed by tumor‐derived or tumor‐associated factors in the microenvironment of regional LN, as nontumor immunoregulatory cell populations that accumulate in sentinel LN or suppressive factors that drain to these sites may create a local milieu that is inhospitable to T cell activation even prior to LN invasion by the tumor. However, even in the case of the progressive B16‐F1 model, we did not observe accumulation of CD4+ CD25+ FOXP3+ regulatory T cells (Tregs) in the lung‐draining LN over the course of tumor progression, and there was no difference in the frequency of these cells in the LN of tumor‐free versus tumor‐bearing mice (Figure S2). Moreover, we considered a role for TGFβ1 in the suppression of CD8+ T cell differentiation, but our recent demonstration that D5.1G4 melanoma does not secrete this immunosuppressive cytokine [25] suggests that this mechanism is not responsible for the failure of anti‐tumor CD8+ T cells to acquire effector function in our model. It of course remains possible that other yet unidentified factors may drain to regional LN ahead of tumor cells and directly suppress CD8+ T cell differentiation within the context of TFLN. In this regard, melanoma‐derived exosomes have been shown to create a pre‐metastatic niche in sentinel LN and support subsequent LN invasion by tumor cells [26], and it has been hypothesized that factors within these exosomes might also impair T cell function [27]. While this possibility remains to be explored in our system, it is interesting to speculate that conditioning of a pre‐metastatic niche within regional LN prior to tumor cell invasion may involve subversion of anti‐tumor immunity through interference with T cell differentiation, either through a direct effect on T cells themselves or through an influence on the immunogenicity of APC. Compromising anti‐tumor immunity through such pre‐metastatic LN conditioning may indeed set the stage for LN seeding by melanoma cells that ultimately acquire the migratory, invasive, and metabolic properties needed to infiltrate and grow in these compartments.

While we continue to explore the influence of melanoma‐derived factors on both T cells and APC populations, we do favor the hypothesis that incomplete differentiation arises from suboptimal T cell stimulation rather than direct T cell suppression, as this idea is consistent with previous work showing that CD8+ T cells do differentiate into functional effectors in B16‐involved LN when they are stimulated by exogenous, CD40L‐activated bone marrow‐derived dendritic cells (BMDC) [14] or when IL‐12 is administered to support T cell responses to tumor‐derived Ag [17]. In this regard, our findings are similar to those of Horton et al., who also found that IL‐12, along with IL‐2, prevented dysfunctional T cell differentiation in their NSCLC model [21]. Moreover, both we and Horton et al. have shown that PD‐1 blockade or combination CTLA‐4/PD‐1 blockade, respectively, fails to support effector T cell differentiation in our models [17, 21], further highlighting incomplete differentiation as a unique form of anti‐tumor T cell dysfunction distinct from exhaustion.

Together, our data have important implications for cancer immunotherapy. Specifically, although the dysfunction of terminally exhausted T cells can be difficult to reverse due to the stability of epigenetically controlled transcriptional programs [28], incomplete CD8+ T cell differentiation may be overcome by strategies that support the immunogenicity of endogenous DC or that employ exogenously activated DC as part of vaccination regimens. In addition to promoting effector CD8+ T cell differentiation against established tumors with IL‐12 or BMDC vaccination as we have previously reported [14, 17], it might also be possible to drive complete effector differentiation of sub‐optimally stimulated T cells by enhancing the immunogenicity of endogenous cross‐presenting DC, either with interventions that promote immunogenic tumor cell death or by using approaches to expand and activate these cells in situ [29]. We are currently investigating how such strategies might influence the differentiation and anti‐tumor reactivity of T cells in our model.

Importantly, because incomplete T cell differentiation may arise early in the course of tumor progression and is not overcome by CTLA‐4 or PD‐1 blockade, this form of immune dysfunction may indeed contribute to the innate resistance to checkpoint blockade therapy exhibited by many cancers. It is interesting to speculate, then, that DC‐based interventions which drive effector T cell differentiation might in turn increase the responsiveness of those cells to checkpoint inhibition, particularly when targeting checkpoints that operate primarily during the effector phase of an anti‐tumor T lymphocyte response. Though this issue has not been specifically investigated in the context of incomplete differentiation to date, others have reported synergistic anti‐tumor effects of DC‐based therapies and checkpoint blockade regimens in preclinical settings [30, 31], and several combinatorial approaches based on these therapies are currently in trial [32, 33].

Finally, while DC‐based approaches for melanoma immunotherapy have yet to be met with the success of checkpoint blockade therapy in the clinic [34, 35], it is important to consider that these strategies may be ideally suited for use at early stages of melanoma progression, even before tumor is detectable in regional LN, as they have the potential to stimulate effector CD8+ T cell differentiation and promote the development of immunologic memory to protect against disease relapse. Although immunizing early‐stage patients with no apparent signs of metastatic disease in order to generate anti‐tumor immune memory is likely not to be appropriate in all cases, such an approach may indeed prove useful for patients whose primary tumors display histologic features or molecular signatures/biomarkers associated with aggressive behavior. Going forward, prospective studies assessing how DC‐based therapies influence the long‐term outcome of such patients will be necessary to determine whether immunologic interventions that induce tumor‐specific effector CD8+ T cell differentiation confer long‐term protection against disease recurrence.

## 5 Conclusions

The incomplete differentiation of tumor Ag‐specific CD8+ T cells is a unique form of anti‐tumor immune dysfunction that may arise early during the course of cancer progression. Lymph node invasion by melanoma cells is not required for the induction of this dysfunctional phenotype, which arises during the priming stage of CD8+ T cell responses to Ag derived from either highly or poorly tumorigenic melanomas.

## Author Contributions

Kristian M. Hargadon was responsible for all aspects of this study, including funding acquisition, project administration, conceptualization, methodology, investigation, validation, formal analysis, resources, writing, visualization, and supervision. Travis B. Goodloe III contributed to funding acquisition, investigation, and validation qPCR studies to assess melanoma cell involvement of lymph nodes. Stephen L. Woodall II contributed to investigation and validation associated with development and characterization of the D5.1G4‐cOVA melanoma cell line.

## Ethics Statement

All experiments were approved by the Hampden‐Sydney College Animal Care and Use Committee and were performed in accordance with regulatory standards and guidelines outlined in the Guide for the Care and Use of Laboratory Animals.

## Conflicts of Interest

The authors declare no conflicts of interest.

## Supporting information


**Figure S1.** Ex vivo analysis of tumor cell outgrowth from LN cultures. Single‐cell suspensions were generated from the paratracheal LN of tumor‐bearing mice and cultured ex vivo to monitor for outgrowth of melanoma cells. Whereas images in Figure 1f were taken at 3 days post‐culture, images shown here for cultures generated from LN of D5.1G4 tumor‐bearing mice were maintained for 10 days, with media replacement occurring every 3 days. Images are representative of cultures from 3 independent experiments, each with 2 mice per group. In no cultures were D5.1G4 melanoma cells cultured out of the population of LN cells, even after this extended ex vivo culture period.

**Figure S2.** Evaluation of regulatory T cells within tumor‐draining lymph nodes. LN harvested from mice as indicated were assessed for the presence of CD4+ CD25+ FOXP3+ Tregs, which did not differ significantly between tumor‐free versus tumor‐bearing animals or between mice bearing tumors at early versus late stages of progression. Plots are gated on CD4+ lymphocytes, and numbers indicate the percentage of CD4+ cells that were positive for both CD25 and FOXP3. Representative plots are shown, and pooled data from 3 independent experiments, each with 2 mice per group, are graphed.

## Data Availability

The data that support the findings of this study are available from the corresponding author upon reasonable request.
