# Supplementary figures and images for: Lymph Node Invasion by Melanoma Cells Is Not Required for the Induction of Incomplete Differentiation by Tumor‐Specific CD8+ T Cells
Source: Cancer Rep (Hoboken). 2025 Feb 10;8(2):e70145. doi: 10.1002/cnr2.70145 (PMC11810983; doi:10.1002/cnr2.70145)

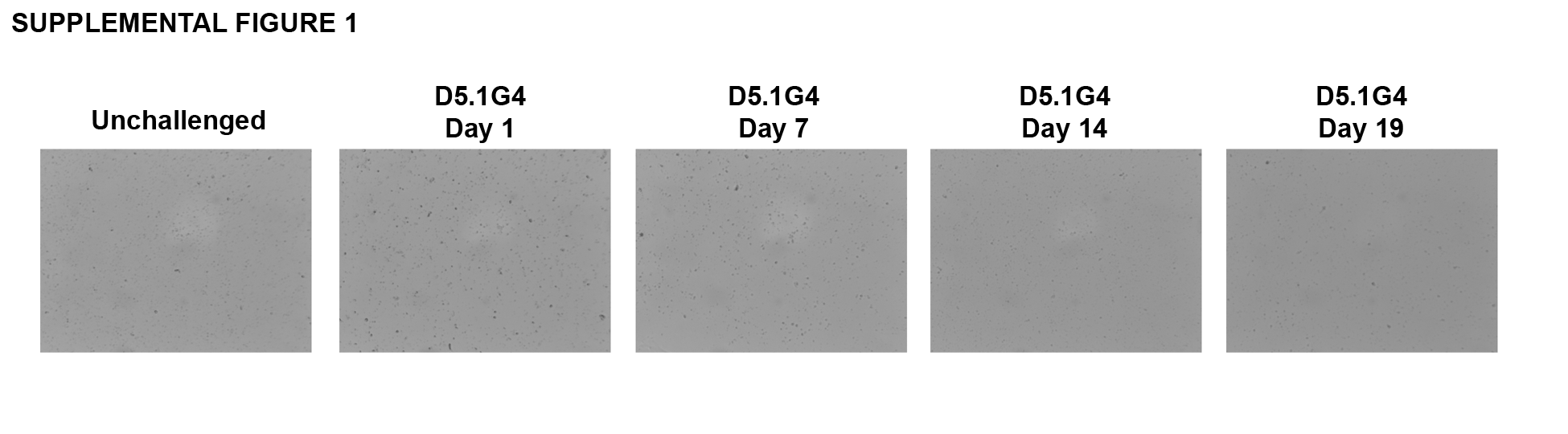

Supplement: Supplementary file 1 — Figure S1. Ex vivo analysis of tumor cell outgrowth from LN cultures. Single‐cell suspensions were generated from the paratracheal LN of tumor‐bearing mice and cultured ex vivo to monitor for outgrowth of melanoma cells. Whereas images in Figure 1f were taken at 3 days post‐culture, images shown here for cultures generated from LN of D5.1G4 tumor‐bearing mice were maintained for 10 days, with media replacement occurring every 3 days. Images are representative of cultures from 3 independent experiments, each with 2 mice per group. In no cultures were D5.1G4 melanoma cells cultured out of the population of LN cells, even after this extended ex vivo culture period. [file CNR2-8-e70145-s002.tif]

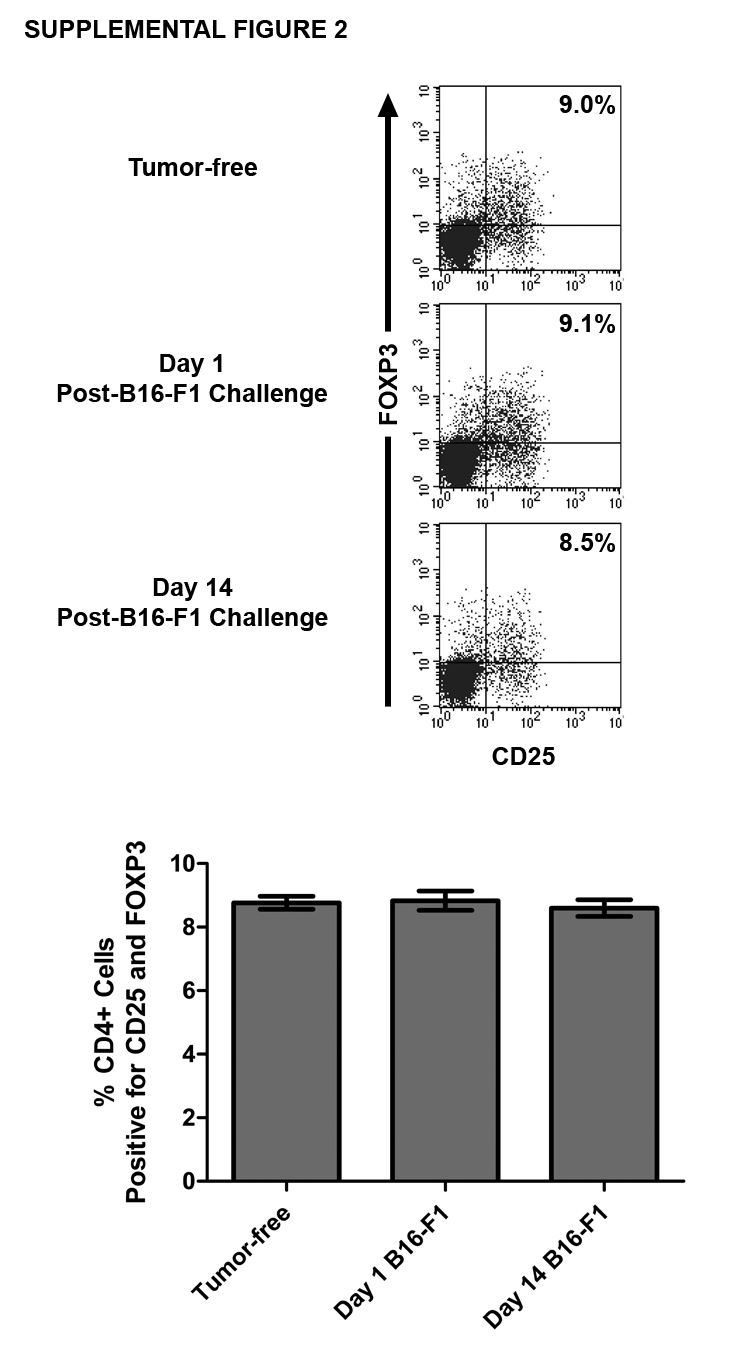

Supplement: Supplementary file 2 — Figure S2. Evaluation of regulatory T cells within tumor‐draining lymph nodes. LN harvested from mice as indicated were assessed for the presence of CD4+ CD25+ FOXP3+ Tregs, which did not differ significantly between tumor‐free versus tumor‐bearing animals or between mice bearing tumors at early versus late stages of progression. Plots are gated on CD4+ lymphocytes, and numbers indicate the percentage of CD4+ cells that were positive for both CD25 and FOXP3. Representative plots are shown, and pooled data from 3 independent experiments, each with 2 mice per group, are graphed. [file CNR2-8-e70145-s001.tif]
